# Oridonin enhances antitumor effects of doxorubicin in human osteosarcoma cells

**DOI:** 10.1007/s43440-021-00324-1

**Published:** 2021-08-24

**Authors:** Liliya Kazantseva, José Becerra, Leonor Santos-Ruiz

**Affiliations:** 1 Andalusian Centre for Nanomedicine and Biotechnology-BIONAND, Universidad de Málaga, Parque Tecnológico de Andalucía, C/ Severo Ochoa, 35, 29590 Campanillas Málaga, Spain; 2 Centro de Investigación Biomédica en Red, Biotecnología, Biomateriales y Nanomedicina (CIBER-BBN), Madrid, Spain; 3 Instituto de Investigación Biomédica de Málaga-IBIMA, Málaga, Spain; 4 Departamento de Biología Celular, Genética y Fisiología Facultad de Ciencias, Universidad de Málaga, Campus de Teatinos, 29071 Málaga, Spain

**Keywords:** Osteosarcoma, Doxorubicin, Oridonin, Synergism, Combination therapy, Cardioprotection

## Abstract

**Background:**

Doxorubicin is the chemotherapeutic drug of choice in osteosarcoma treatment, but its cumulative administration causes dilated cardiomyopathy. Combination therapy represents a potential strategy to reduce the therapeutic dosage of the chemotherapeutic agent and minimize its side effects. The aim of this study was to evaluate the potential of oridonin, a natural product from the medicinal herb *Rabdosia rubescens*, to act in combination with doxorubicin for osteosarcoma treatment. To date, there are no reports of the simultaneous administration of both drugs in osteosarcoma therapy.

**Methods:**

The combined administration of different doses of oridonin and doxorubicin, as compared with the drugs alone, were tested in an *in vitro* model of osteosarcoma. The synergistic effect of the drugs on cell death was assessed by *alamarBlue*^*™*^ and by *CompuSyn* software. Early and late apoptosis markers (JC-1 fluorescence and Annexin V immunofluorescence), as well as the production of reactive oxygen species, were evaluated by flow cytometry. Western blot was used to assess the expression of anti-apoptotic proteins.

**Results:**

Oridonin and doxorubicin presented a synergistic cytotoxic effect in osteosarcoma cells. In the presence of sub-cytotoxic concentrations of the natural product, there was an increased accumulation of intracellular doxorubicin, increased levels of reactive oxygen species (ROS), alteration of mitochondria membrane potential and a higher rate of apoptosis.

**Conclusion:**

The combined use of oridonin and doxorubicin could help to reduce the clinical dosage of doxorubicin and its dangerous side effects.

## Introduction

Osteosarcoma is a bone tumor characterized by the presence of differentiated osteoblasts producing immature osteoid matrix. It is one of the most common cancers affecting children and adolescents, and has a second peak of incidence after the age of 50 [1, 2].

Doxorubicin (DOX) is possibly the most commonly used drug alone or in combination with high-dose methotrexate and cisplatin for osteosarcoma treatment [2, 3]. It is an anthracycline that achieves its therapeutic activity through DNA intercalation, leading to inhibition of topoisomerase-II function. Its efficiency, however, is limited by presence of a life-threatening side effect in the form of cardiotoxicity, which depends on cumulative dosing and can drive to congestive heart failure later in life [2, 4–6].

There is a growing interest in combination therapy that is based on administration of two or more drugs acting on different target sites, making it difficult for cancer cells to mutate and adapt to novel conditions. The synergistic effects observed between different drug types may drive to a reduction of drug dosing, toxicity, resistance, and to an increase of therapeutic efficiency [2, 7–9].

Oridonin (ORI) is a diterpenoid isolated from *Rabdosia rubescens*, (Hemsl.), H.Hara, a medicinal herb that has been used to treat esophageal cancer by native people of Henan province, in China [10, 11]. This natural product is getting much attention for having anti-angiogenic properties and ability to inhibit growth and metastasis in different types of cancer, such as liver, colorectal and breast [12–15]. There are very few studies about the possibilities of ORI in osteosarcoma treatment, but these have shown promising results [16–18]. Synergy was found in combination with DOX against aggressive breast cancer, suggesting that the combined use of both drugs could help to decrease therapeutic DOX doses, thus reducing as well its secondary effects, like cardiotoxicity [15].

In the present study, we aimed to gain insight on the potential of a DOX plus ORI combination therapy for osteosarcoma treatment, by focusing on an *in vitro* model of pediatric osteosarcoma. We found that DOX and ORI possess a synergistic effect, as co-administration of both drugs significantly increased osteosarcoma cell death as compared with the drugs administered alone. Our data point to the possibility of using ORI as an adjunct in DOX-based chemotherapy treatments for osteosarcoma.

## Materials and methods

### Reagents

Unless otherwise stated, all reagents were purchased from Sigma-Aldrich (St. Louis, Missouri, USA).

### Cell culture

Saos-2 cell line, an osteogenic sarcoma derived from a primary osteosarcoma of an 11-year-old girl, was purchased from the European Collection of Authenticated Cell Cultures (ECACC 89,050,205) [19]. It was maintained in McCoy’s 5A medium, supplemented with 15% heat-inactivated Fetal Bovine Serum (FBS), and 2 mM L-glutamine. It was incubated at 37 °C in a humidified atmosphere with 5% CO_2_ (standard culture conditions).

### Cell viability

Cell viability was assessed using *alamarBlue*^*™*^ assay from Invitrogen (Carlsbad, California, USA). Cells were seeded at a density of 20,000 cells/well in 100 µL of the corresponding medium, in a 96-well plate. To let cell attachment, the plate was incubated in standard culture conditions for 24 h. After this time, Saos-2 was treated with different concentrations of DOX (0.1–10 µM), or ORI (10–40 µM), administered in the culture medium. After 48 h of incubation, the treatment medium was removed and the cells were rinsed with Phosphate Buffered Saline (PBS). Cell viability was measured by incubating the cells for 4 h in 20% *alamarBlue*^*™*^ dissolved in culture medium. Then, the *alamarBlue*^*™*^ fluorescence emission was measured on a *LS55* fluorometer plate reader (Perkin Elmer, Waltham, Massachusetts, USA) at 530 nm-excitation and 590 nm-emission wavelengths. The obtained data allowed selecting the concentration that achieved 50% of cell death (CD_50_) for each drug. Then, combined drug therapy was evaluated by treating cells with different combinations of DOX (CD_50_, CD_50_/2 and CD_50_/10) and ORI (CD_50_, CD_50_/2 and CD_50_/10) and assessing viability as described. Six wells were used for each condition in all experiments. Experiments were repeated thrice.

The interaction between DOX and ORI was quantified through the Chou-Talalay method [20]. For this, the combination index (CI) was calculated using the *CompuSyn* software (ComboSyn, Inc., Paramus, New Jersey, USA), where CI < 1 indicated synergism, CI = 1 additive effect and CI > 1 antagonism.

### Cell uptake of DOX

To establish if there are changes in the intracellular accumulation of DOX due to the presence of ORI, intracellular DOX fluorescence was measured by flow cytometry. For this purpose, Saos-2 cells were seeded at a density of 150,000 cells/well in 12-well plates and incubated for 24 h in standard culture conditions to allow cell attachment. Then, Saos-2 cells were treated for 2 h with 2.5 µM DOX, 10 µM ORI and 2.5 µM DOX + 10 µM ORI. These concentrations were chosen based on their calculated CIs. Untreated cells were used as control. The treatment conditions were performed in triplicate. After incubation, cells were rinsed with PBS, collected by trypsinization and centrifuged at 1800 rpm for 5 min. The supernatant was removed and the pellet resuspended in 1 mL of cold PBS with 2% of heat-inactivated FBS. Then, the cells were transferred to 5 mL tubes and analyzed with a *Gallios* flow cytometer (Beckman Coulter, Brea, California, USA). DOX fluorescence was detected in FL3 channel. For each sample 25,000 events were collected. Experiments were repeated thrice.

### Cytotoxicity-related cellular events

To understand how the drugs under study induced cytotoxicity, mitochondrial membrane potential, apoptosis, and reactive oxygen species were assayed. For this purpose, Saos-2 cells were seeded into 12-well multi-well plates, at a density of 150,000 cells/well, in normal medium, and incubated for 32 h in standard culture conditions. Then, they were treated for 16 h with 2.5 µM DOX, 10 µM ORI and combined DOX + ORI (2.5 µM and 10 µM, respectively). After drug exposure, the treatment medium was removed and cells were washed with PBS, collected by trypsinization and pelleted by centrifugation at 1800 rpm for 5 min. The pellet was then treated as described below for each assay. Cells without any treatment were used as controls. Each condition was done in triplicate. Experiments were replicated thrice.

#### Mitochondrial membrane potential

Changes of the mitochondrial inner membrane electrochemical potential were detected with *Mitochondria Staining Kit*, according to the manufacturer’s instructions. Briefly, pelleted cells were resuspended in 1 mL of JC-1 staining mixture, (5 µg/mL JC-1 dissolved in staining buffer, according to the manufacturer’s instructions), and incubated for 20 min, in standard culture conditions. Cells treated with valinomycin were used as a control for mitochondrial gradient dissipation. For this purpose, valinomycin (0.2 µg/mL final concentration) was added to the JC-1 staining mixture. After incubation in JC-1 staining solution, the cells were pelleted by centrifugation, the supernatant removed, and the pellet resuspended in ice-cooled JC-1 staining buffer. The fluorescence of the stained cells was measured by flow cytometry. JC-1 monomers were detected in the FL1 channel and JC-1 aggregates were detected in the FL2 channel. For each sample 10,000 events were collected.

#### Apoptosis assay

Apoptosis was detected by flow cytometry using *Annexin V-FITC/PI Apoptosis Detection kit* (Elabscience, Houston, Texas, USA). Manufacturer’s instructions were followed with some modifications. Presence of DOX inside the cells was overlapping with PI in FL3 channel, leading to the overestimation of the dead cells. For this reason, Hoechst 33258 was used instead. Pelleted cells were resuspended, and incubated for 20 min, in 100 µL Annexin V binding buffer containing 2.5 µL Annexin V-FITC conjugate and 2.5 µL Hoechst staining solution. Then, 400 µL binding buffer was added and cells were transferred to FC tubes to be analyzed by flow cytometry. For each sample 10,000 events were collected. The fluorescence of Annexin V-FITC was detected in FL1 channel, and Hoechst 33258 in FL9. Cells in the lower left quadrant (Annexin V-FITC − /Hoechst 33258 −) were considered to be live cells. Early apoptotic event (Annexin V-FITC + /Hoechst 33258 −) was considered when cells were located in the lower right quadrant. Late apoptotic event (Annexin V-FITC + /Hoechst 33258 +) was considered if cells localized in the upper right quadrant. Finally, necrosis (Annexin V-FITC − /Hoechst 33258 +) was considered when cells were localized in the upper left quadrant. The apoptotic index was calculated by combining the data of early and late apoptosis. Data were normalized to the control, which percentage of apoptosis was considered as 100%.

#### Reactive oxygen species (ROS) assay

The intracellular ROS level was measured by flow cytometry using the *Fluorometric Intracellular ROS kit*. Manufacturer instructions were followed with some modifications. Pelleted Saos-2 cells were resuspended in 500 µL of assay buffer and 1 µL 500X ROS detection reagent stock solution was added to each tube. The cells were incubated for 1 h at 37 °C in a humidified atmosphere with 5% CO_2_. Fluorescence intensity was measured in the FL6 channel of a flow cytometer. For each sample, 10,000 events were collected.

### Western Blot analysis

Cells were harvested with a scraper, and lysed in RIPA lysis buffer containing a mix of protease inhibitors. Bradford protein assay was used to determine the total protein concentration. Proteins were separated on SDS-PAGE and transferred onto nitrocellulose membrane. Non-specific antibody binding was blocked with 5% non-fat milk. The membrane was incubated with the following primary antibodies: anti-Mcl-1 (1:1000), anti-Bcl-2 (1:1000), anti-Bcl-XL (1:1000) and anti-*β*-actin (1:2000) overnight at 4 °C. Subsequently, a horseradish peroxidase-conjugated secondary antibody (1:5000) was incubated at room temperature for 2 h. Finally, the protein bands were visualized with ECL Western Blotting Substrate and photographed with ChemiDoc XRS + (Bio-Rad). Experiments were performed thrice. Semi-quantification of band densitometry was carried out by measuring band density with *ImageJ* software. All bands in a gel were normalized against the gel background, and then Mcl-1, Bcl-2 and Bcl-XL bands were normalized against their corresponding ß-actin loading controls.

### Statistical analysis

Quantitative data were graphed and statistically analyzed with *Excel 16.0* (ed. 2016, Microsoft, Redmond, Washington, USA), and *Prism 6.0c* (ed. 2013, GraphPad Software Inc., San Diego, California, USA). Shapiro–Wilk test was used for normality evaluation of the data. The results were analyzed by a two-way analysis of variance (ANOVA) followed by Tukey’s multiple comparison test. Values with *p *< 0.05 were considered statistically significant. Data are represented as mean ± SD. Western-blot semi-quantitative data were analyzed by Kruskal–Wallis test.

## Results and discussion

**Fig. 1:**
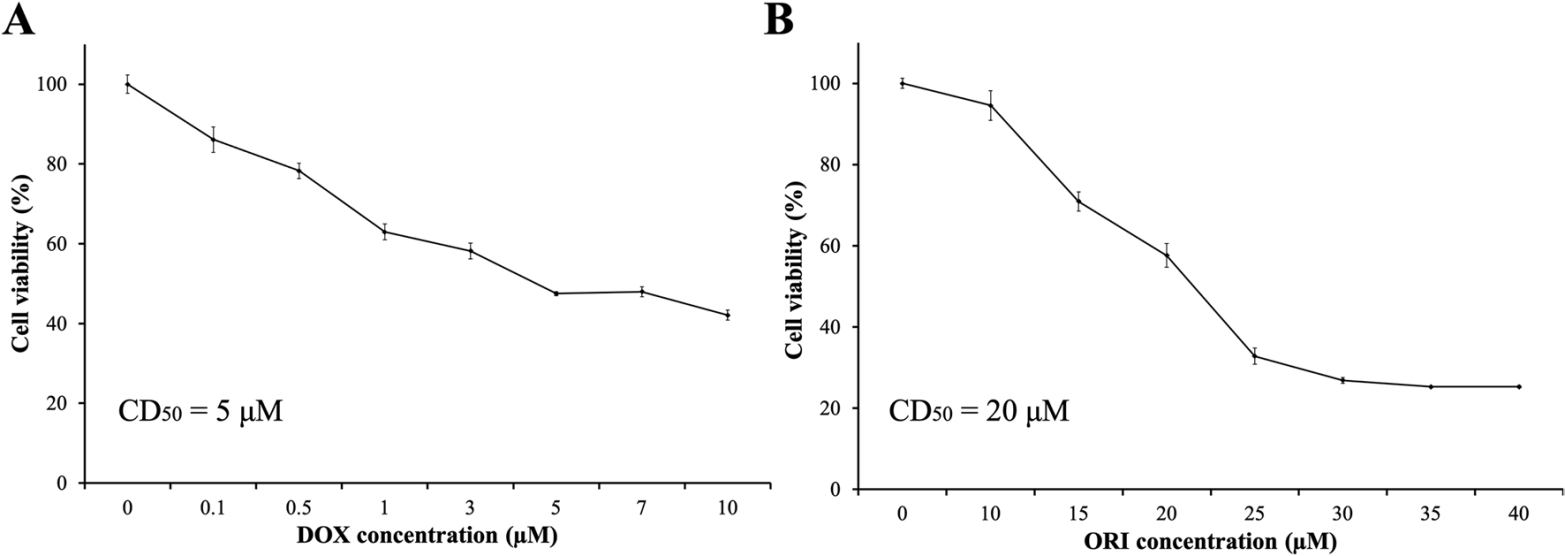
Toxicity evaluation of DOX and ORI. Saos-2 viability was assessed after 48 h of exposure to different concentrations of DOX (**A**) and ORI (**B**) using *alamarBlue*^*™*^ assay. Data are represented as mean ± SD

In the present study, we aimed to evaluate the possibility of enhancing the effect of DOX, a common drug in the treatment of osteosarcoma, by combining it with ORI. This combination has not been studied yet for this type of cancer. Saos-2 cells were treated with 7 different concentrations of DOX and ORI in order to select the concentration that achieved a 50% of cell death (CD_50_) for each drug after 48 h of exposure. Our results confirmed that both drugs, individually administered, reduced cell viability in a dose-dependent manner (Fig. 1A and B). DOX showed a more toxic effect than ORI, with the drug concentration causing 50% cell death being determined as CD50 ≈ 5 µM for DOX, and CD50 ≈ 20 µM for ORI.

**Fig. 2:**
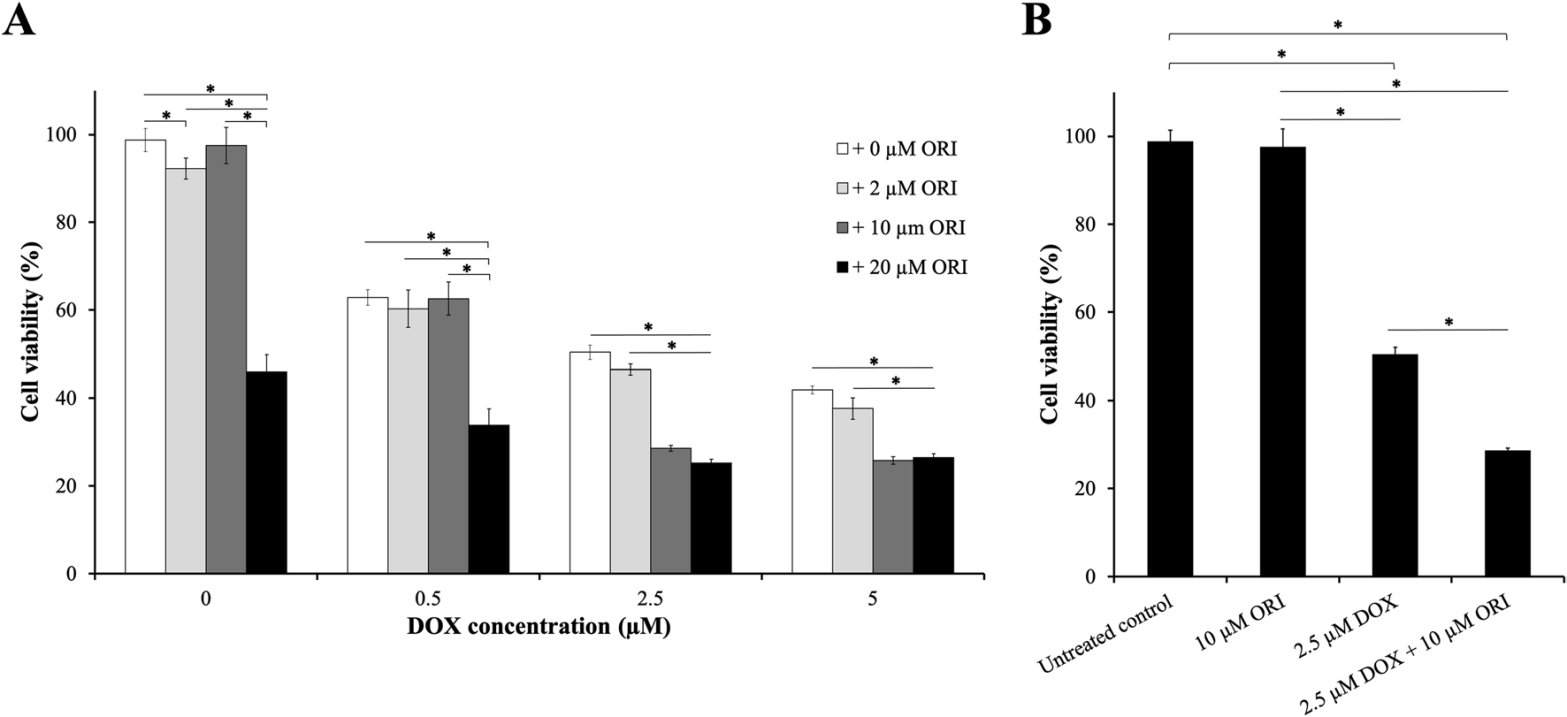
Toxicity evaluation of DOX and ORI combination. Saos-2 cells were exposed for 48 h to concentrations of DOX and ORI corresponding to their CD_50_, CD_50_/2 and CD_50_/10; as well as to combinations of both drugs (**A**). Separate representation of the selected concentration of DOX, ORI and combination of both chosen for the posterior experiments (**B**). Cell viability was assessed by *alamarBlue*^*™*^ assay. Analysis was done using two-way ANOVA test followed by Tukey’s multiple comparisons test. Asterisk denotes statistical significance. Significant effect was observed with two-way ANOVA: *F*_3,80_ = 680.9, *p *< 0.0001 for main effect and *F*_9,80_ = 100.6, *p *< 0.0001 for interaction. In 0 µM DOX group, the post hoc analysis indicated significance in the following subgroups: *p *= 0.0004 between 0 µM ORI and 2 µM µM ORI; *p *= 0.0074 between 2 µM ORI and 10 µM ORI; *p *< 0.0001 in the rest of the subgroups. In 0.5 µM DOX, the post hoc analysis indicated significance in the following subgroups: *p *< 0.0001 in subgroups with the asterisk. In 2.5 µM DOX, the post hoc analysis indicated significance in the following subgroups: *p *= 0.0464 between 0 µM ORI and 2 µM µM ORI; *p *< 0.0001 in the rest of the subgroups. In 5 µM DOX, the post hoc analysis indicated significance in the following subgroups: *p *= 0.0255 between 0 µM ORI and 2 µM µM ORI; *p *< 0.0001 in the rest of the subgroups. Data are represented as mean ± SD

**Fig. 3:**
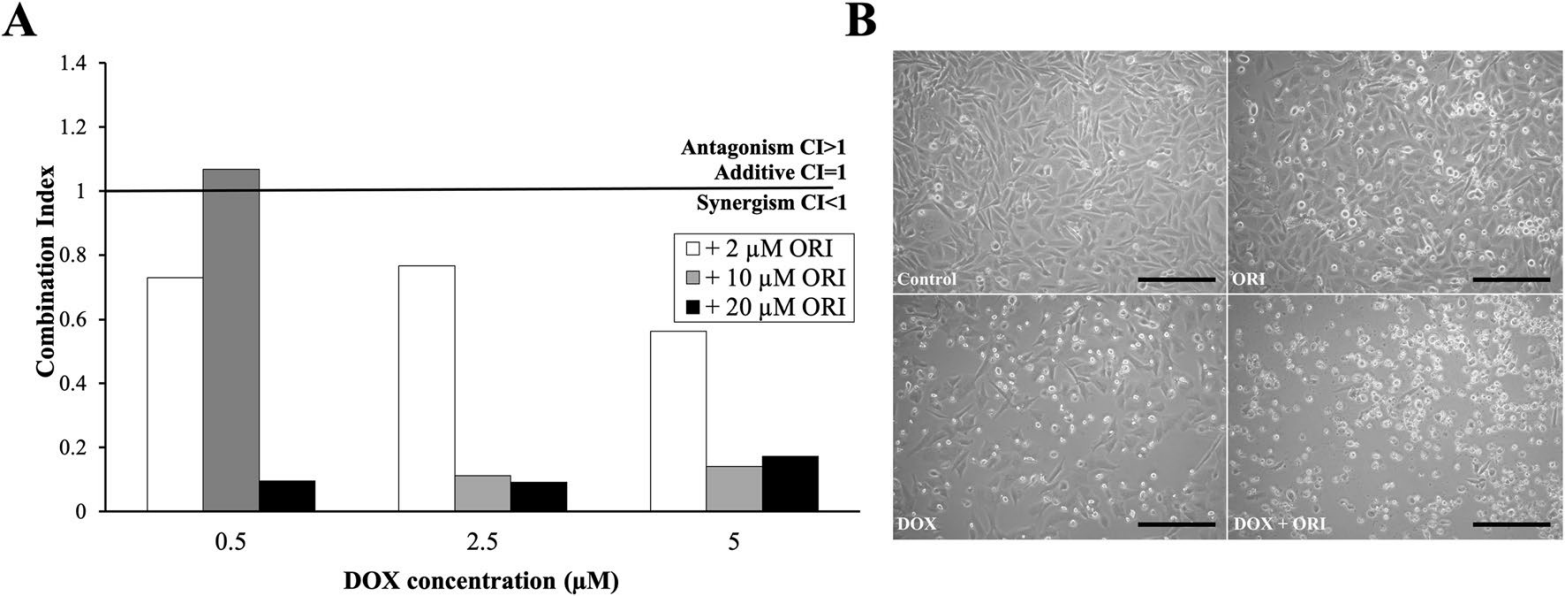
Effects of ORI, DOX and the combination of both on Saos-2. Combination Index (**A**). Morphology of Saos-2 cultures after 16 h of treatment with 2.5 µM DOX, 10 µM ORI, and the combination of both was observed using a 10X objective in an inverted microscope (**B**). Scale bar = 50 μm

Based on the cytotoxicity induced by the drugs alone, the effects of different DOX doses (CD_50_, CD_50_/2 and CD_50_/10) (CD_50_, CD_50_/2 and CD_50_/10) combined with different doses of ORI (CD_50_, CD_50_/2 and CD_50_/10) were evaluated. In general, simultaneous treatment produced a higher cytotoxic effect compared to drugs alone (Fig. 2A). The synergistic effect of DOX and ORI in Saos-2 cytotoxicity was confirmed by calculating the Combination Index (CI; Fig. 3A), which is a quantitative definition of synergism, additive and antagonism [20]. These results thus confirm that combination with ORI would allow an increased chemotherapeutic effect of DOX, and might then represent a step forward for the reduction of DOX unwanted side effects.

**Fig. 4:**
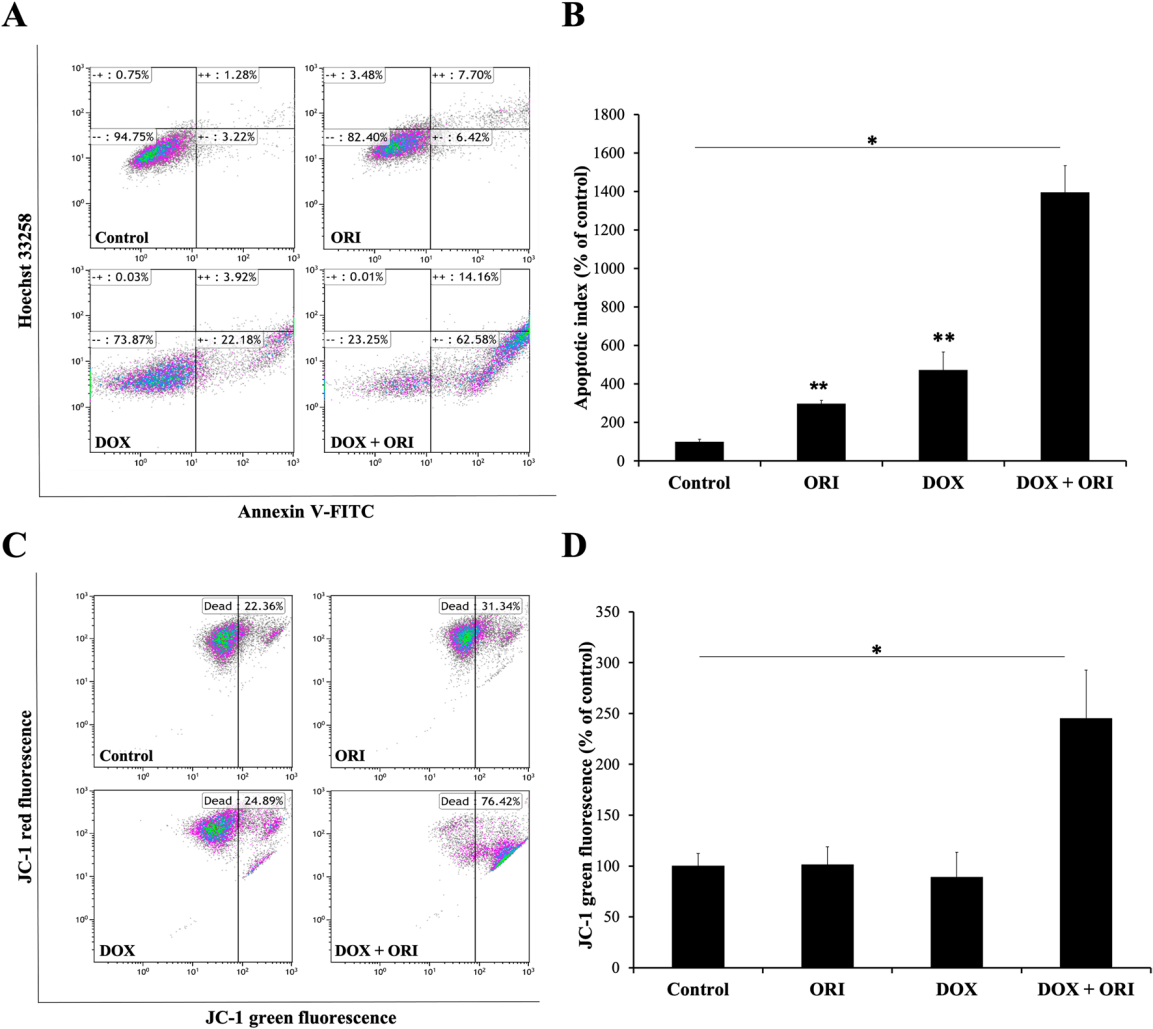
Effects of ORI, DOX and the combination of both on Saos-2 cell death. Annexin V-FITC and Hoechst 33258 fluorescent signals, as measured by flow cytometry (**A**). Apoptotic index as determined by Annexin V-FITC/Hoechst 33258 staining (**B**). In each group, the data of early and late apoptosis were combined and normalized to the control, which percentage of apoptosis was considered as 100%. Significance was observed with two-way ANOVA (*F*_3,6_ = 217.2; *p *< 0.0001). Post hoc analysis indicated significant effect in the following groups: *p *< 0.0001 in DOX + ORI *vs*. Control/ORI/DOX; *p *= 0.0432 in Control *vs.* ORI; *p *= 0.0021 in Control *vs*. DOX. JC-1 red and green fluorescent signals in each experimental group, as measured by flow cytometry (**C**). Mitochondrial membrane potential as determined by quantification of JC-1 fluorescence. Significance was observed with two-way ANOVA (*F*_3,6_ = 30.01; *p *= 0.0005). Post hoc analysis indicated significant effect in the following groups: *p *= 0.0011 in DOX + ORI *vs.* Control; *p *= 0.0012 in DOX + ORI *vs.* ORI; *p *= 0.0008 in DOX + ORI *vs.* DOX (**D**). One asterisk shows statistical significance between DOX + ORI and the rest of the treatment groups, while two asterisks is compared to the control (ORI/DOX *vs.* Control) according to a two-way ANOVA test followed by Tukey’s multiple comparison test. Data are represented as mean ± SD

We decided to carry on our experiments with 2.5 μM DOX + 10 μM ORI. This dosing corresponds to a twofold reduction over DOX CD_50_ (Fig. 2B), combined with a minimally cytotoxic dose of ORI (Figs. 2B shows that cells treated with 10 µM ORI are almost 100% viable). After 16 h of treatment, the selected DOX + ORI pair presented enhanced mortality, with only few cells still adhered to the plate, as compared to the rest of the controls (Fig. 3B). We could confirm that apoptosis, as checked by Annexin V-FITC/Hoechst 33258 staining, was induced in cultures by DOX and ORI alone, where the apoptotic index was significantly higher in cells treated with the combined drugs (Fig. 4A and B).

From previous studies in osteosarcoma, it is known that ORI induces apoptosis through mitochondrial toxicity [17]. Accordingly, after 16 h of treatment, significant changes in mitochondrial potential were observed in Saos-2 cells treated with DOX + ORI, where a marked switch from red to green fluorescence indicated a mitochondrial membrane depolarization, which is an early feature of cell death (Fig. 4C and D) [21].

**Fig. 5:**
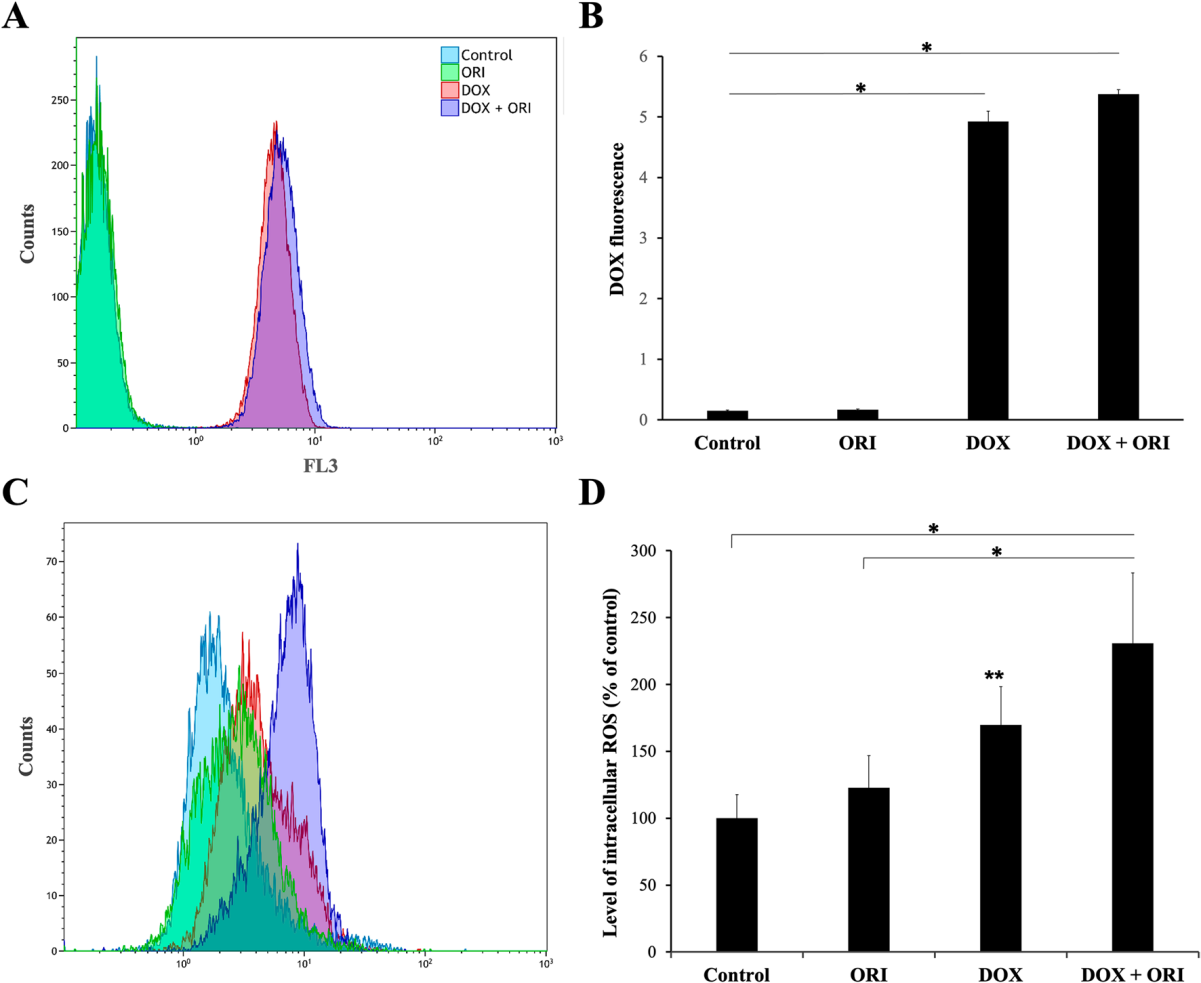
Effects of ORI, DOX and the combination of both on Saos-2. DOX fluorescent signal in each experimental group, as measured by flow cytometry (**A**). Quantification of DOX red fluorescence signal in control and drugs-treated Saos-2 cells after 2 h of treatment. One asterisk shows statistical significance between DOX/DOX + ORI and the rest of the treatment groups. Significant effect was observed with two-way ANOVA (*F*_3,6_ = 2589; *p *< 0.0001). Post hoc analysis indicated significance in the following groups: *p *< 0.0001 in DOX + ORI *vs.* Control/ORI and *p *= 0.0057 in DOX + ORI *vs.* DOX; *p *< 0.0001 in DOX *vs.* Control/ORI (**B**). ROS fluorescent signal in each experimental group, as measured by flow cytometry (**C**). Quantification of intracellular ROS levels (**D**). One asterisk shows statistical significance between DOX + ORI and Control/ORI, while two asterisks is DOX compared to the Control. Significant effect was observed with two-way ANOVA (*F*_3,6_ = 18.13; *p *= 0.0021). Post hoc analysis indicated significance in the following groups: *p *= 0.0020 in DOX + ORI *vs.* Control; *p *= 0.0054 in DOX + ORI *vs.* ORI; *p *= 0.0414 in DOX *vs.* Control (**D**). Analysis was done using two-way ANOVA test followed by Tukey’s multiple comparison test. Data are represented as mean ± SD

To understand how ORI enhanced the toxicity of the chemotherapeutic drug, DOX cell uptake was studied using flow cytometer, on account of the red fluorescence of DOX. After 2 h of exposure to DOX plus ORI, Saos-2 cells presented a greater shift in DOX fluorescence as compared to DOX alone, indicating that ORI had increased the intracellular uptake of DOX (Fig. 5A and B). Hence, it is reasonable to hypothesize that intracellular DOX accumulation induced by ORI might have caused mitochondrial toxicity and this, in turn, produced cellular apoptosis. In general, our results are in accordance with the findings of Li et al*.* where synergy of ORI was found in combination with DOX against aggressive breast cancer, suggesting that the combined used of both drugs could help to decrease therapeutic DOX doses, thus reducing as well its secondary effects, such as cardiotoxicity [15].

We further investigated the possible mediator that could be implicated in the triggered apoptosis. It is known DOX induces apoptosis through ROS induction, even in cells like Saos-2 that lack functional p53 [22]. Additionally, cancer cells are characterized by elevated levels of oxidative stress, meaning that chemotherapeutic agents that can promote a further increase in ROS production are a preferential strategy against cancer [23]. To determine its participation in mitochondrial-mediated cell death, we studied intracellular ROS levels in Saos-2 osteosarcoma cells. Both DOX and ORI were able to increase intracellular ROS levels, as compared to untreated controls (Fig. 5C and D). These results point to ROS induction being responsible for the activation of the cascade leading to cell death in Saos-2 cells, as has been observed in other studies [22, 24].

**Fig. 6:**
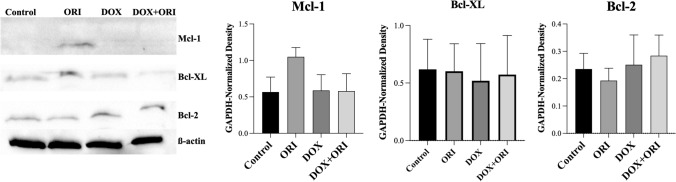
Effects of ORI, DOX and the combination of both on the expression of apoptosis-related proteins in Saos-2. The expression of Mcl-1, Bcl-2 and Bcl-XL was detected after Saos-2 were exposed to DOX + ORI for 16 h. *β*-actin was used as internal reference. The gel images depict the more representative blots out of three experiments. The graphs represent protein densitometry measured in the three gels as mean ± SD. Data were analyzed by a Kruskal–Wallis and Dunn’s multiple comparisons test

Then, we studied the effects of each treatment on the expression of Mcl-1, Bcl-2 and Bcl-XL, anti-apoptotic members of the Bcl-2 protein family, by Western Blot analysis (Fig. 6). Mcl-1 was only detected in cells exposed to ORI alone. Bcl-2 and Bcl-XL were observed in both control and experimental conditions, being their level lower in DOX + ORI-treated cells than in cells treated with DOX alone. This can account for the higher level of apoptosis observed in DOX + ORI-treated cells, as compared with those that received the chemotherapeutic drug alone. In contrast, ORI exposed cells presented reduced induction of ROS and less cell death, suggesting a link between oxidative stress and apoptosis in our model. These results agree with the higher levels of Mcl-1, Bcl-2 and Bcl-XL observed in ORI-treated cells. Demelash et al*.* found that Mcl-1 expression prevented ROS formation, through inhibition of NOX4, a NADPH oxidase, and its posterior translocation to the mitochondria [25]. In our model, Mcl-1 expression could be promoting the survival of Saos-2 cells treated with low doses of ORI. Possibly, higher doses would inhibit the activation of Mcl-1 leading to apoptosis, as observed from Fig. 6. However, further studies will be needed to confirm and elucidate the mechanism of ROS implication in all the experimental groups.

In this study, our results confirmed that combination with ORI would allow both an increased chemotherapeutic effect of DOX, and a drastic reduction of its therapeutic dose. In a breast cancer model the simultaneous administration of both drugs induced apoptosis by reducing the expression of Bcl-2 and Survivin, while increasing the expression of Bax. Moreover, DOX and ORI combination was shown to suppress angiogenesis possibly through the inhibition of VEGFR2-mediated signaling pathway [15]. Several studies demonstrated ORI to enhance the cytotoxicity of different chemotherapeutic drugs that could be explained by more than one mechanism and the cellular pathways affected could be specific or shared between different types of tumors. In hepatocellular carcinoma, a synergistic effect was observed between cisplatin and ORI, which was produced by inhibition NF-κB transcription, one of the major regulators of innate and adaptive immunity [26]. In gastric cancer ORI was reported to reverse cisplatin resistance by suppressing P-gp expression, which is involved in drug efflux [27].

In summary, our results provide a first insight of DOX plus ORI combination therapy for osteosarcoma treatment. The simultaneous administration of both drugs induced apoptosis through mitochondrial toxicity, possibly mediated by ROS. By increasing DOX cytotoxic effect in osteosarcoma cells, ORI could allow reducing DOX doses in osteosarcoma treatment.

## Abbreviations

DOX: Doxorubicin
ORI: Oridonin
ROS: Reactive Oxygen Species
CI: Combination Index
CD_50_: 50% Cell Death
